# Supplementary material for: Entosis: From Cell Biology to Clinical Cancer Pathology
Source: Cancers (Basel). 2020 Sep 1;12(9):2481. doi: 10.3390/cancers12092481 (PMC7563411; doi:10.3390/cancers12092481)
Supplement: Supplementary file 1 [file cancers-12-02481-s001.pdf]

cancers

Review

Entosis: from cell biology to clinical cancer pathology

Izabela Mlynarczuk-Bialy, Ireneusz Dziuba, Agnieszka Sarnecka, Emilia Platos, Magdalena Kowalczyk, Katarzyna K. Pels, Grzegorz M. Wilczynski, Cezary Wojcik and Lukasz P. Bialy

Supplementary data: Protocol S1

Cell culture: BxPC3 pancreatic cancer cells (ATCC) were cultured in RPMI1640 medium supplemented with 10% FBS, stable glutamine and antibiotic antimycotic solution (all from Gibco). Cells were kept in a humidified atmosphere containing 5% CO_2 at 37 °C and were passaged every 3–4 days with a standard 0.25% trypsin/EDTA solution (Gibco).

For analysis of culture conditions suitable for entosis, BxPC3 cells were cultured in TC treated flasks or at non-treated Petri dishes or in the presence/absence of EDTA (1, 5, 10 μM).

For confocal microscopy, BxPC3 cells were cultured on histological glass slides-cell culture chambers, BD354108, (Becton Dickinson). After 12 h from seeding, cells were stained for 15 min with Vybrant™ CFDA SE Cell Tracer Kit V12883 (Promega, Madison, WI, USA) according to manufacturers' instructions. Next, cells were washed with PBS, fixed in ice-cold buffered 70% ethanol for 2 min, washed 3 × 5 min with PBS and mounted with DAPI-VectaShield (Vector Laboratories). Specimens were observed by means of Leica SP5 confocal microscope with Las AF software (Leica).

For classical light microscopy observations, cells were maintained similarly as mentioned above including fixation step with 70% cold buffered ethanol. Then specimens were subjected to staining with Haematoxylin Solution, Harris Modified for 5 min followed by standard histological routine and subsequently were mounted in DPX medium (all from Sigma/Merck). Delta Optical light microscope with camera was used for observations.
